# Sensorimotor control: computing the immediate future from the delayed present

**DOI:** 10.1186/s12859-016-1098-2

**Published:** 2016-07-25

**Authors:** Arman Sargolzaei, Mohamed Abdelghani, Kang K. Yen, Saman Sargolzaei

**Affiliations:** 1 Department of Electrical and Computer Engineering, Florida International University, Miami, FL 33174 USA; 2 Department of Mathematics and Statistics, University of Alberta, Edmonton, 30332 Canada; 3 Research and Development Department, Rancs Group LLC, Miami, FL 33172 USA

**Keywords:** Sensorimotor control, Time delay estimation, Vestibulo-Ocular Reflex (VOR), Predictive control, Motor learning

## Abstract

**Background:**

The predictive nature of the primate sensorimotor systems, for example the smooth pursuit system and their ability to compensate for long delays have been proven by many physiological experiments. However, few theoretical models have tried to explain these facts comprehensively. Here, we propose a sensorimotor learning and control model that can be used to (1) predict the dynamics of variable time delays and current and future sensory states from delayed sensory information; (2) learn new sensorimotor realities; and (3) control a motor system in real time.

**Results:**

This paper proposed a new time-delay estimation method and developed a computational model for a predictive control solution of a sensorimotor control system under time delay. Simulation experiments are used to demonstrate how the proposed model can explain a sensorimotor system’s ability to compensate for delays during online learning and control. To further illustrate the benefits of the proposed time-delay estimation method and predictive control in sensorimotor systems a simulation of the horizontal Vestibulo-Ocular Reflex (hVOR) system is presented.

Without the proposed time-delay estimation and prediction, the hVOR can be unstable and could be affected by high frequency oscillations. These oscillations are reminiscent of a fast correction mechanism, e.g., a saccade to compensate for the hVOR delays. Comparing results of the proposed model with those in literature, it is clear that the hVOR system with impaired time-delay estimation or impaired sensory state predictor can mimic certain outcomes of sensorimotor diseases. Even more, if the control of hVOR is augmented with the proposed time-delay estimator and the predictor for eye position relative to the head, then hVOR control system can be stabilized.

**Conclusions:**

Three claims with varying degrees of experimental support are proposed in this paper. Firstly, the brain or any sensorimotor system has time-delay estimation circuits for the various sensorimotor control systems. Secondly, the brain continuously estimates current/future sensory states from the previously sensed states. Thirdly, the brain uses predicted sensory states to perform optimal motor control.

## Background

Sensorimotor control system is the most robust and versatile collection of modular, hierarchical and well-organized hybrid of control strategies. With it we can land a plane, drive a car, play sports, and adapt to bizarre sensorimotor transformations [1, 2] (e.g., reversing prisms), or we are allowed to perform in space or in the deep sea. Sensorimotor control system can accomplish all of these tasks in an optimal manner in terms of speed, accuracy and efficiency [3, 4].

The brain’s sensorimotor cortex, as a complex neural sensorimotor control system, inherently finds and implements an optimal decision to a vast range of input conditions. Noises, nonlinearities, delays, uncertainties and redundancies are among the major problems that the sensorimotor control system interacts with [5]. Delays occur in various parts of a sensorimotor control system, ranging from sensory information reception to, information transmission along nerve fibers, computing responses by processing the sensory information, feedback transmission, and finally, motor output in terms of muscle reaction. The delay value is dynamic and varies with sensory modality. For example there is longer delay for vision than proprioception. This is because the complexity of the sensory information processing depends on the task and it is longer for face recognition than motion perception. Efferent control signals are delayed as a result of neural conduction delays and low-pass filtering properties of muscles. Short efferent delays, such as the monosynaptic Stretch Reflex (SR), are in the order of 10–40 milliseconds, depending on the length and the type of nerve fiber. This delay is increased to 30–70 milliseconds for the cortical component of the long latency SR [6]. The electromechanical delay, such as the delay in generating force response in muscles, can take up to 25 milliseconds [7]. So far, the investigation focuses on how a complex neural sensorimotor control system such as the brain’s sensorimotor cortex is capable of performing tasks in the presence of the above-mentioned conditions. However, the study in this paper specifically focuses on developing a mathematical model that will simulate a sensorimotor control system in the presence of noise and delay.

The Vestibulo-Ocular Reflex (VOR), one of the fastest involuntary responses due to the short neural connections and high neural computation speed, is not prone to the occurrence of delay up to 10 milliseconds from stimulus onset [8]. VOR is a motor control system that stabilizes vision during head movements. Smooth pursuit, another efficient visual control system in human beings for target tracking in their visual field, has the ability to process the information with a 80–130 millisecond delay in the brain [9, 10]. Delays make control difficult because information about the current state of the motor system is outdated. A motor control system that does not have delay compensation mechanisms could not correct for errors, leading to potential inefficiencies and instability. The explanation of those controllers such as fuzzy logic control, feedback and adaptive linearization based control, optimal nonlinear trajectory control can be found in the literature [11–13]. Although they have several applications but they should be modified to be used in systems with time delays.

### Delay compensation: facts and current theories

Consider the saccadic eye movement, which is a fast eye movement produced by a visual system that directs the eyes to interesting visual stimuli: the movement duration is shorter than the sensory delay [9]. This means that sensory feedback about the current state of the eye and the visual field cannot be used to correct or guide saccades because the sensory information regarding the movement itself arrives after the completion of the movement. Smooth pursuit eye movements allow a person to track targets in the visual field at a high speed of ~200°/sec, which is a remarkable performance. Recent experiments stated the high performance of the smooth pursuit system, where it was observed that the position of eyes was ahead of visual sensory feedback of the target position. This cannot be achieved by solely implementing standard negative feedback methods based on visual error signals [14–16].

Under such information processing and transmission delays, simple feedback control is affected by significant temporal discrepancies between target signal and current state, suggesting that some form of predictive control must take place to achieve such a high performance [17]. The predictive nature of sensorimotor control systems is explored and demonstrated through multiple experiments. Experiments demonstrated that monkeys have the ability to conduct smooth pursuit movements with zero retinal slip [18, 19] or the ability to maintain smooth pursuit during blink periods (momentary disappearances of the target) [20]. Such predictive compensation was observed both in tracking moving targets with constant velocity or in sinusoidal moving objects. In a hand movement study, it was demonstrated that the cerebellum is involved in predicting the position of the hand during a movement [21]. The predicted state of the limb from the history of motor commands allows the motor control to act on this estimate of state rather than relying solely on a delayed sensory feedback. This suggests that cerebellar output is a signal that can be combined with delayed sensory feedback elsewhere in the brain in order to generate real-time state estimates for motor control.

### Time-delay estimation and control

A primate’s sensorimotor controller is equipped with the ability to predict motor movements, as well as possess the ability to compensate for time delays. Time-delay estimation is a difficult problem, as it renders even the simplest linear systems nonlinear, yet biological control systems are robust enough to deal with time delays. However, it is not known how this is achieved. Current time-delay estimation techniques mainly cover linear systems, including: constant time delays, random time delay with specific noise characteristics, or restricted dynamic time delay [9, 10, 22–27]. However, most biological systems exhibit some degree of variability, nonlinearity, and uncertainty, which may make above mentioned methods developed inapplicable. Furthermore, most delay estimation procedures are not used in the context of predictive control methodology. The Hilbert-Huang Transform-based method is found to be the most efficient delay estimation technique with a focus on practical applicability to the motor control; however, the process is a complex one [10].

A comprehensive computational model to explain time-delay compensation in biological control is lacking. The study in this paper proposes a sensorimotor learning and control model that estimates variable time delays, predicts sensory states from delayed sensory feedback, and controls a motor system in real time. Accurate models of sensorimotor control systems result in a better understanding of the function of the human sensorimotor cortex, with practical applications in understanding the mechanisms underlying neurological disorders such as autism [28] and epilepsy [29].

The next section covers the proposed time-delay estimation method and develops a computational model for a predictive control solution for a sensorimotor control system under time delay. The proposed model is evaluated in a real time, with online learning and control simulation processes. The paper is concluded with findings and suggestions for future research.

## Methods

Suppose the sensorimotor system can be approximated in a region of interest by the linear time-varying system, as stated in Eq. (1):

1 $$ \overset{.}{x}(t)=A(t)x(t)+B(t)u(t) $$

where *x*(*t*) is the state vector (e.g., the position of the eye or hand in space, etc.), *u*(*t*) is the control vector or the neural motor commands (e.g., the firing of motor-neurons or muscle contractions, etc), and *A*(*t*) and *B*(*t*) are time-varying matrices with appropriate dimensions. The matrix *A*(*t*) represents the influence of the current state *x*(*t*) of the motor system to its future changes $\overset{.}{x}(t)$. The matrix *B*(*t*) is the sensorimotor controller gain, which determines how motor commands affect $\overset{.}{x}(t)$. It is common for *A*(*t*) and *B*(*t*) to change over time. Examples include joint friction, viscosity and elasticity of muscles, etc. All of which change over time.

The solution to the first order differential Eq. (1) is given by:

2 $$ x(t)={e}^{{\displaystyle {\int}_0^tA}(s)ds}{x}_0+{\displaystyle \underset{0}{\overset{t}{\int }}{e}^{{\displaystyle {\int}_s^tA}(v)dv}B(s)u(s)ds} $$

where *x*_0_ is the initial state [30].

Let $G(t)={e}^{{\displaystyle {\int}_0^tA}(s)ds}$ and Eq. (2) written in terms of *G(t)* is

3 $$ x(t)=G(t){x}_0+G(t){\displaystyle \underset{0}{\overset{t}{\int }}{G}^{-1}(s)B(s)u(s)ds} $$

where *x*(*t*) is the current state of the sensorimotor system measured by the sensor organs. The motor command vector is *u*(*t*). Motor commands are usually sensed at the level of the effector by specialized sensory organs. For example, muscle spindles measure the force generated in the muscle and communicate the information to the brain. Here, we assume that *x*(*t*) and *u*(*t*) are precisely measured by sensory organs.

Suppose the sensory time-delay vector is represented by **τ** = [*τ*_*i*_] (*i*^th^ time delay value). For simplicity we assumed *τ* = *τ*_*i*_ in the rest of this paper. The solution of Eq. (2) with the time delay is

4 $$ \begin{array}{l}x\left(t-\tau \right)=G\left(t-\tau \right){x}_0\\ {}\kern2.64em +G\left(t-\tau \right){\displaystyle \underset{0}{\overset{t-\tau }{\int }}{G}^{-1}(s)B(s)u(s)ds}\end{array} $$

Before proceeding with a solution for the time-delay problem and an associated predictive control method in the brain, let us state our assumption about the representation of time in the brain. We assume that the brain is a truly autonomous system. In other words, there are no clocks in the brain that count the ticks of *absolute time*. All sensation of time is the result of externally perceived periodic stimuli. This is unlike industrial control systems, where there are synchronized clocks that count the ticking of time and the time variable *t* can be accessed directly. Direct access to a time variable *t* is not possible in the brain’s sensorimotor control system.

We assume the brain keeps an internal estimate of time delays, denoted as $\widehat{\boldsymbol{\uptau}}$. The error signal is calculated as $\xi =x\left(t-\tau \right)-x\left(t-\widehat{\tau}\right)$, where *x*(*t* − *τ*) is the delayed sensory signal. The delayed sensory signals are known to the brain, but the brain cannot access the time-delay vector ***τ*** directly. On the other hand, $x\left(t-\widehat{\tau}\right)$ is unknown since $\widehat{\boldsymbol{\uptau}}$ is unknown. However, *x*(*t* − *τ*) can be computed from the knowledge of *G(t)*, *B(t)* and *u(t)*.

To compute $\widehat{\boldsymbol{\uptau}}$, a modified version of the gradient descends method is used:

5 $$ \frac{d\widehat{\tau}}{dt}=-\eta \zeta \frac{\partial \zeta }{\partial \widehat{\tau}} $$

wheres *η* is the learning parameter.

Using Eq. (3), we can express Eq. (5) in a form,

6 $$ \begin{array}{l}\frac{d\widehat{\tau}}{dt}=-\eta \zeta \frac{\partial \left[x\left(t-\tau \right)-x\left(t-\widehat{\tau}\right)\right]}{\partial \widehat{\tau}}=\eta {e}_m\frac{\partial x\left(t-\widehat{\tau}\right)}{\partial \widehat{\tau}}\\ {}\kern0.72em =\eta \zeta \frac{\partial G\left(t-\widehat{\tau}\right)}{\partial \widehat{\tau}}\left[{x}_0+{\displaystyle \underset{0}{\overset{t-\widehat{\tau}}{\int }}\left(B(s)/G(s)\right)u(s)ds}\right]\\ {}\kern1.8em -\eta \zeta \left\{B\left(t-\widehat{\tau}\right)u\left(t-\widehat{\tau}\right)-G\left(t-\widehat{\tau}\right)B(0)u(0)\right\}\end{array} $$

The time delay *τ* can be estimated using Eq. (6). However, there are biological constraints that need to be considered. Equation (6) requires the knowledge of $x\left(t-\widehat{\tau}\right)$, $G\left(t-\widehat{\tau}\right)$ and *u* for any $0\le \widehat{\tau}\le t-\tau$. But, this is impossible because it needs to store the full history of motor commands *u*(*t*) or all functions, *G(t)* and *x(t)*. Therefore, assuming the biological plausibility of Eq. (6) without boundedness assumptions on the maximum delay *τ* is not possible.

To guarantee stability and limited memory usage, the following condition, *τ* ≤ *τ*_*max*_ is added. This condition is reasonable and does not in any way limit the generality of the method. Furthermore, most human movements are either repetitive, such as walking, or intermittent with many pauses, such as reaching. In reaching, at the beginning of the movement, the initial position of the arm is known, and the delay is not an issue because the arm is at rest. At the end of the movement, the arm is coming back to rest and the final state of the arm is known. Therefore, delays have no detrimental effects. However, during the motion, the state of the arm keeps on changing which causes the values communicated to the brain with variable delays. It is during the arm’s motion that the delay estimation is paramount. Since movements are finite in time, applying a limit on the maximum number of delays is reasonably justified.

In terms of hardware implantation, it is necessary to store the history of constructed signals in a finite buffer. Actually, the brain automatically stores history about signals like *u*(*t*) and *x*(*t*)*.* One possible scenario is for the brain to learn the dynamics of *G*(*t*) and *B*(*t*) and thereby compute the dynamics of *x* and *u* for any time period.

Here, we assume that the brain stores *u*(*t*) from *t* to *t* − *τ*_*max*_, as well as *G*(*t*), *B*(*t*) and *x*(*t*). It should be noted that if delays exceed *τ*_*max*_, a complete open-loop control prevails.

The most important in the proposed control system is to predict the future state of a sensorimotor system, given that the delayed state and an estimate of the time delay are known. To do so, Eqs. (3) and (4) are combined, as follows:

7 $$ \begin{array}{l}x(t)=G(t){G}^{-1}\left(t-\tau \right)x\left(t-\tau \right)\\ {}\kern1.56em +G(t){\displaystyle \underset{t-\tau }{\overset{t}{\int }}B(s){G}^{-1}(s)u(s)ds}.\end{array} $$

Then, the state can be predicted using estimated time-delay, $\widehat{\tau}$, as follows

8 $$ \begin{array}{l}\widehat{x}(t)=G(t){G}^{-1}\left(t-\widehat{\tau}\right)x\left(t-\widehat{\tau}\right)\\ {}\kern1.56em +G(t){\displaystyle \underset{t-\widehat{\tau}}{\overset{t}{\int }}B(s){G}^{-1}(s)u(s)ds}.\end{array} $$

It should be noted that *x*(*t* − *τ*) is what is actually measured and delivered to the sensorimotor plant model in the brain, represented by Eq. (4). However, *G*(*t*) and the integral over *u*(*t*) are both dependent on the estimate of the time delay $\widehat{\tau}$. When the error in the estimate of time delay $\varepsilon =\widehat{\tau}-\tau$ decreases to zero, the predicted state approaches to the actual state *x*(*t*).

The next step involves finding a way to combine the sensorimotor plant model with the motor controller. Let the difference between the desired sensory goal *r*(*t*) and current state *x*(*t*) be the performance error *e*(*t*) = *r*(*t*) − *x*(*t*), and the estimate of the performance error be $\widehat{e}(t)=r(t)-\widehat{x}(t)$. Here, we define a PID controller input in terms of the estimated error as [31, 32]:

9 $$ u(t)={K}_P\widehat{e}(t)+{K}_D\frac{d\widehat{e}(t)}{dt}+{K}_I{\displaystyle \underset{0}{\overset{t}{\int }}\widehat{e}}(s)ds $$

and the optimal feedback controller as

10 $$ u(t)=K\widehat{e}(t) $$

where *K*_*P*_, *K*_*D*_, *K*_*I*_ and *K* are proportional gain, derivative gain, integral gain and optimal gain, respectively.

The PID controller and the optimal feedback controller gains can be designed as if there was no delay with information about the predicted state. Essentially, the controller depends on the error *ê*(*t*) that results from the estimate $\widehat{x}(t)$. So, if the estimate $\widehat{x}(t)$ converges to *x*(*t*), then *ê*(*t*) converges to *e*(*t*).

**Fig. 1:**
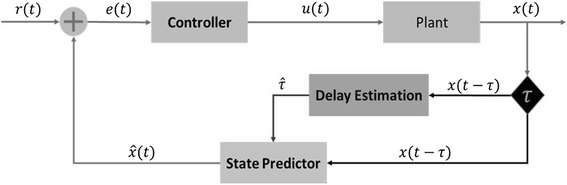
Model of the sensorimotor control with time-delay estimation and sensory states predictor. The Plant Box symbolizes a higher level of a sensorimotor system such as the eye, associated muscles, sensors, responses, goals and objectives (e.g., minimize retinal slip in VOR). The Controller Box is the neural network responsible for achieving the required task in an “optimal” way. Delay Estimator is the circuit we are proposing as the brain, or a sensorimotor systems to estimate time delay in a sensory and motor pathway. Sate Predictor is where current and “future” sensory states are estimated and predicted, respectively, based on the estimated current time delay. The “*r*(*t*)” is the desired goal, or in the language of control theory, the reference trajectory. The “*e*(*t*)” is the error difference between the desired sensory state and predicted sensory state $\widehat{x}(t)$. The “*u*(*t*)” are the motor commands to muscles or effector organs. The *x*(*t* − *τ*) are the delayed sensory states. The “*x*(*t*)” is the actual sensory state. Finally, “τ” is the time delay that could either be a natural time delay or a consequence of damage or disease

Next is the recap of the concept about sensorimotor time-delay estimation, state prediction and control. Figure 1 shows the elements and connectivity between components of the proposed computational model of a brain sensorimotor control system at a higher level. It is assumed that the brain wiring is a way to carry out the computations in accordance to the schematics in Fig. 1.

Tables 1 and 2 show the lists of known and unknown sensorimotor control variables of the brain, respectively. Three main assumptions in the proposed computational model for the brain sensorimotor control system are: 1) the brain possesses a time-delay estimator circuit; 2) the brain uses the estimate of time delay to predict the current state; and 3) the brain uses the current predicted estimate to control motor movements.

**Table 1:** Sensorimotor control known variables

Variable	Definition
*r*(*t*)	Goal or reference
*x*(*t* − *τ*)	Delayed sensory inputs
*ê*(*t*)	Prediction errors
$\widehat{\tau}$	Estimated time delay
$G\left(t-\widehat{\tau}\right)$	Estimated sensory Jacobian
*B*	Control Jacobian
*u*	Motor commands

**Table 2:** Sensorimotor control unknown variable

Variable	Definition
*τ*(*t*)	Real time delay
*x*(t)	Current sensorimotor state

To illustrate the benefits of the time-delay estimation method of predictive control in the sensorimotor system, we have chosen to simulate the VOR system. The method has been implemented with MATLAB R2013a.

## Results and Discussion

In the horizontal Vestibulo-Ocular Reflex (hVOR), *x* ∈ *ℝ* is the eye position relative to the head, and *u* ∈ *ℝ* is the net motor-neuron signal to the horizontal eye muscles. So, the hVOR system equation in its simplest form [1] is shown as:

11 $$ \overset{.}{x}=-\frac{\kappa }{\rho }x+\frac{1}{\rho }u $$

where *κ* is the coefficient of viscosity and ρ is the coefficient of elasticity, and both are constants. The retinal-image slip velocity is *y* ∈ *ℝ*, which is the sum of eye and head velocities,

12 $$ y=\overset{.}{x}+\overset{.}{h} $$

The goal of the hVOR is to make the retinal slip equal to zero, i.e., *y =* 0. Here, the reference signal *r* is *-h* and the feedback error signal e is *x* - *r* or *x* + *h*. Therefore, $y=\overset{.}{e}$_,_ and the feedback control law is basically a derivative control given by

13 $$ u(t)={K}_D\overset{.}{e}(t) $$

Choosing the appropriate *K*_*D*_ results in $\overset{.}{e}=y=0$.

With sensory delay ***τ***, the measured state of the hVOR control system will be *x*(*t* − *τ*) instead of *x*(*t*), which means a form of time-delay estimation and a plant state predictor.

Based on our formulation, the time-delay estimator can be written as

14 $$ \begin{array}{l}\overset{.}{\widehat{\tau}} = \frac{\eta }{\rho}\zeta \left[{e}^{-\left(\kappa /\rho \right)\left(t-\widehat{\tau}\right)}\left(\rho {x}_0+{\displaystyle \underset{0}{\overset{t-\widehat{\tau}}{\int }}{e}^{-\left(\kappa /\rho \right)\left(s-\widehat{\tau}\right)}u(s)ds}\right)\right]\\ {}\kern0.96em -\frac{\eta }{\rho}\zeta u\left(t-\widehat{\tau}\right)\end{array} $$

where it is assumed that *u*(0) = 0. The state predictor can be found as

15 $$ \widehat{x}(t)={e}^{\left(\kappa /\rho \right)\widehat{\tau}}x\left(t-\tau \right)+\frac{e^{-\left(\kappa /\rho \right)t}}{\rho }{\displaystyle \underset{t-\widehat{\tau}}{\overset{t}{\int }}{e}^{\left(\kappa /\rho \right)s}u(s)ds} $$

**Fig. 2:**
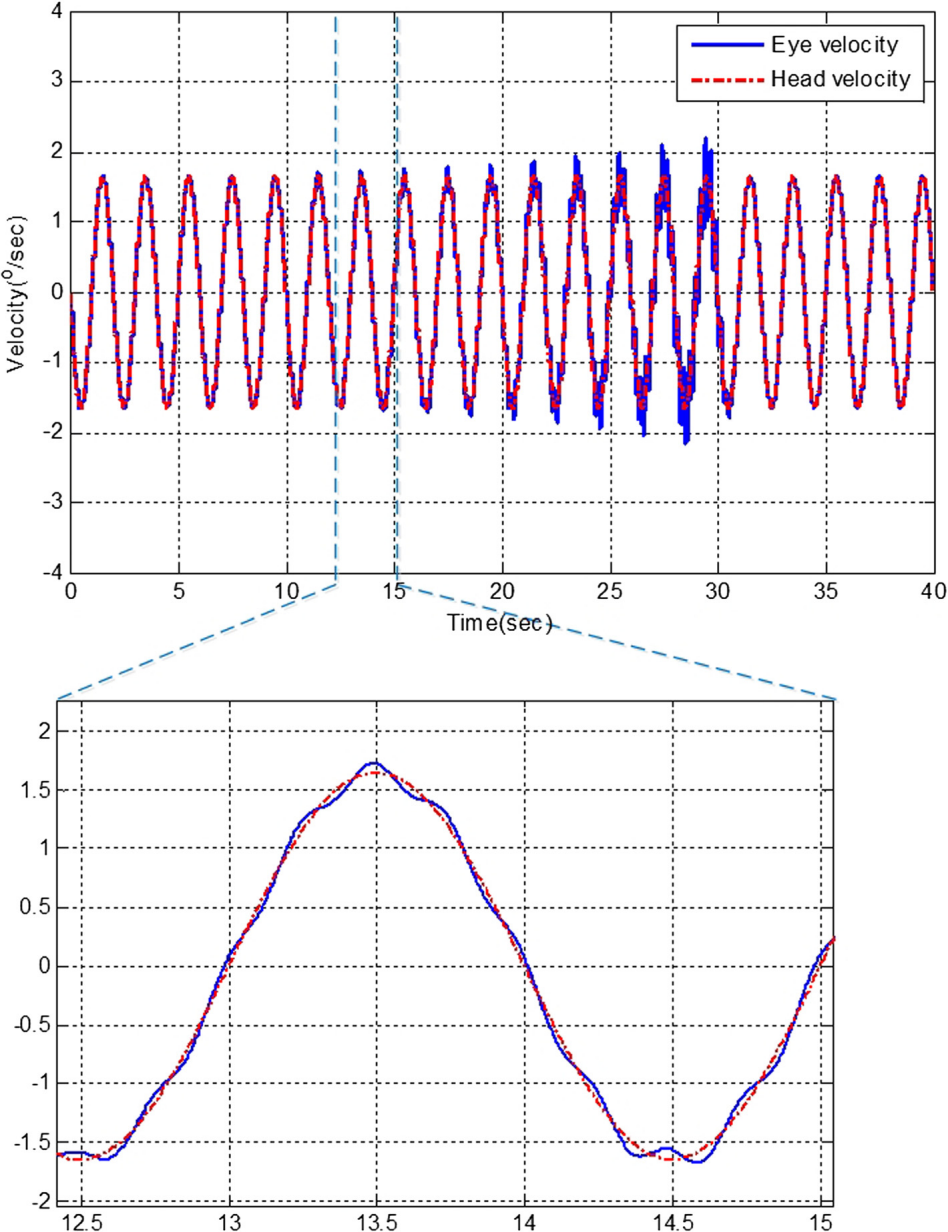
The hVOR performance with failed time-delay compensation, traditional methods. The figure shows a poor response of the eye rotation to head rotation with traditional controller or. The eye is responding to a delayed head velocity. And as result, the eye velocity (*Blue solid line*) is oscillating around the head velocity (*Red dashed line*), see expanded view. This oscillatory behavior is as if the eye is executing a corrective movement (saccades) to compensate for the delay head velocity value. However, it often overshoots the target head velocity. This oscillatory behavior continues until the hVOR fails to do its job completely. The same behavior in Figure could also be the result of a damaged sensory state predictor. In this case even if the time-delay estimation is working properly the state predictors fails to predict the correct current state. As a result the hVOR will be plagued with oscillations and instability

Without time-delay estimation and prediction, the hVOR is unstable and could be affected by high frequency oscillations (see Fig. 2). These oscillations are reminiscent of a fast correction mechanism, e.g., a saccade to compensate for hVOR delays [33].

**Fig. 3:**
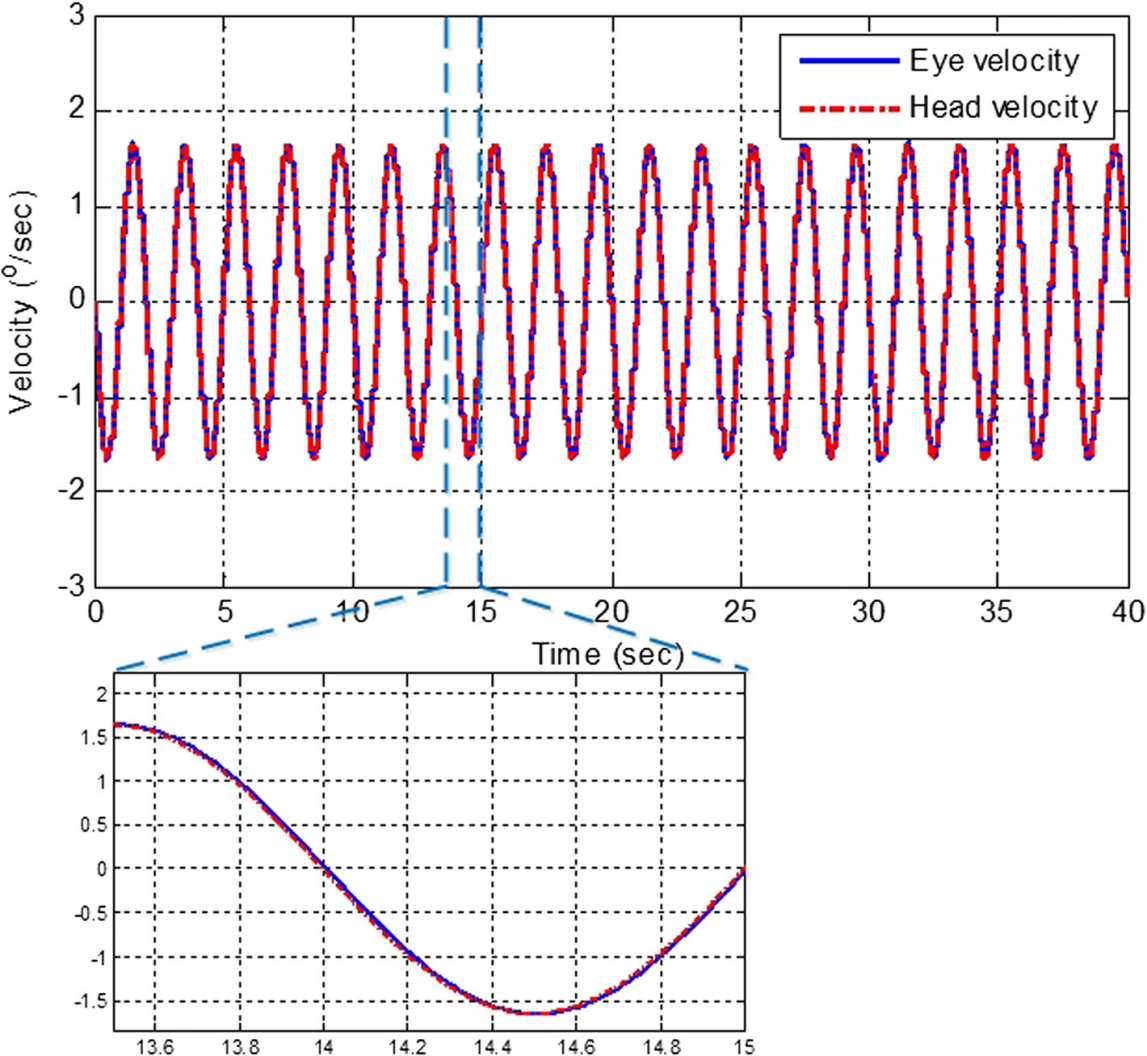
The hVOR performance with existence of time-delay estimator and state predictor under long time delay. The hVOR performance under long time delay (*10 ms*) with the proposed method. The hVOR system is stable when the brain model is equipped with time-delay estimator and state predictor. The figure clearly shows that the hVOR is performing as it should be. The eye velocity is the reverse of the head velocity

Comparing our simulation result in Fig. 2 with that in “Fig. 3” of reference [34]. It clearly shows that the hVOR system with impaired time-delay estimation or impaired sensory state predictor can mimic certain outcomes of sensorimotor diseases.

**Fig. 4:**
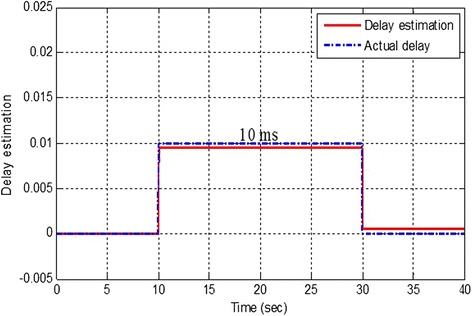
The response of the time-delay estimation circuit for the hVOR system. Blue dashed line is the variation of the time delay during horizontal head movement. Red solid line is the simulated tracking of the time-delay estimation circuit

However, if the control of hVOR is augmented with a time-delay estimator (Fig. 4) and a predictor for eye position relative to the head, then hVOR control is stable and smooth (see Fig. 3).

## Conclusion

In this paper, we have proposed a sensorimotor learning and control model that can predict the dynamics of variable time delays and the future sensory states from the delayed sensory information; learned new sensorimotor realities; and controlled motor system in real time. The results have demonstrated that our developed model can explain the ability of a sensorimotor system compensating delays during real-time control.

This development boils down to three claims, with varying degrees of experimental support. Firstly, we claim that the brain or sensorimotor systems possess time-delay estimation circuits. Secondly, the brain continuously estimates current/future sensory states from the previously sensed states. Thirdly, the brain uses predicted sensory states to perform optimal motor control. Essentially, this process requires performing feedback control by using predicted states.

The work makes further predictions that the brain does not need to use any form of clocking mechanism to sync various aspects of motor control systems affected by delays. In other words, the brain is a data-driven asynchronous collection of sensorimotor control systems. Also, fast and random perturbations to the motor control systems cannot be predicted and may cause instability. The predictive nature of the primate sensorimotor system and its ability to compensate for long delays have been shown by several behavioral and physiological experiments.
